# Lipoxin A_4_ encapsulated in PLGA microparticles accelerates wound healing of skin ulcers

**DOI:** 10.1371/journal.pone.0182381

**Published:** 2017-07-28

**Authors:** Mouzarllem Barros Reis, Priscilla Aparecida Tartari Pereira, Guilherme Ferreira Caetano, Marcel Nani Leite, Alyne Fávero Galvão, Francisco Wanderley Garcia Paula-Silva, Marco Andrey Cipriani Frade, Lúcia Helena Faccioli

**Affiliations:** 1 Departamento de Análises Clínicas, Toxicológicas e Bromatológicas, Faculdade de Ciências Farmacêuticas de Ribeirão Preto, Universidade de São Paulo, Ribeirão Preto, São Paulo, Brazil; 2 Departamento de Clínica Médica, Divisão de Dermatologia, Faculdade de Medicina de Ribeirão Preto, Universidade de São Paulo, Ribeirão Preto, São Paulo, Brazil; Universidade Federal do Rio de Janeiro, BRAZIL

## Abstract

Lipoxin A_4_ (LXA_4_) is involved in the resolution of inflammation and wound healing; however, it is extremely unstable. Thus, to preserve its biological activities and confer stability, we encapsulated LXA_4_ in poly-lactic-co-glycolic acid (PLGA) microparticles (LXA_4_-MS) and assessed its application in treating dorsal rat skin lesions. Ulcers were sealed with fibrin adhesive and treated with either LXA_4_-MS, unloaded microparticles (Un-MS), soluble LXA_4_, or PBS/glue (vehicle). All groups were compared at 0, 2, 7, and 14 days post-lesions. Our results revealed that LXA_4_-MS accelerated wound healing from day 7 and reduced initial ulcer diameters by 80%. Soluble LXA_4_, Un-MS, or PBS closed wounds by 60%, 45%, and 39%, respectively. LXA_4_-MS reduced IL-1β and TNF-α, but increased TGF-β, collagen deposition, and the number of blood vessels. Compared to other treatments, LXA_4_-MS reduced inflammatory cell numbers, myeloperoxidase (MPO) concentration, and metalloproteinase-8 (*MMP8*) mRNA in scar tissue, indicating decreased neutrophil chemotaxis. In addition, LXA_4_-MS treatment increased macrophages and IL-4, suggesting a positive impact on wound healing. Finally, we demonstrated that WRW4, a selective LXA_4_ receptor (ALX) antagonist, reversed healing by 50%, indicating that LXA_4_ must interact with ALX to induce wound healing. Our results show that LXA_4_-MS could be used as a pharmaceutical formulation for the treatment of skin ulcers.

## Introduction

Wound healing is a complex process in which tissues are repaired post injury. Wound healing is divided into inflammatory, proliferative, and remodeling phases [1] that result in scar formation. Scarring involves recruitment of cells, growth factors, cytokines, and eicosanoids along with the release of enzymes to elicit extracellular matrix formation [1,2]. Pathological alterations of each phase are related to wound exacerbation or an inefficient healing process [3]. The aforementioned phases are potentially ideal pharmacological targets that may enhance wound healing [4]. Identifying effective drugs and/or therapies is critically important since complications in wound healing incurs a cost of more than $25 billion annually [5].

Lipid mediator lipoxins (LXs) are eicosanoids derived from arachidonic acid (AA) metabolism. Lipoxygenases 5, 12, and 15-lipoxygenase (LO) generate LXs via interactions with leukocytes and other cells, like platelets and epithelial cells [6,7]. LXs were described in 1984 when 15-hydroxyeicosatetraenoic acid (H(p)ETE), an AA metabolite, was added to human leukocytes. The most abundant compound contained four double conjugated bonds and was subsequently named LXA_4_ [8]. It was later shown that LXA_4_ binds to a specific receptor named ALX, which is a G protein-coupled receptor and is expressed in a variety of cells [7].

After receptor binding to cell types such as neutrophils and macrophages, LXA_4_ induces various responses, including the resolution of inflammation and wound healing [9–13]. In polymorphonuclear cells (PMN), LXA_4_ reduces pro-inflammatory functions by inhibiting reactive oxygen species (ROS) production and nuclear factor kappa-light-chain-enhancer of activated B cells (NF-κB) and activator protein-1 (AP-1) activation as well as impairing PMN transmigration across the vessel [7]. In monocytes, LXA_4_ enhances macrophage phagocytosis of apoptotic PMN [14], creating a wound healing microenvironment at inflammation sites [7]. Furthermore, LXA_4_ is endogenously produced in the eye to induce a faster wound healing response in the cornea [15], and reduces the release of inflammatory mediators by human epidermal keratinocytes [16]. Based on the abovementioned reasons, LXA_4_ is heralded as a critical resolutive and wound healing mediator [9,10,12,13,15]. To this end, we hypothesized that LXA_4_ also promotes wound healing in the skin. We employed PLGA (poly-lactic-co-glycolic acid) microparticles (MS) as a means to preserve LXA_4_ biological activities and to promote controlled release, taking into consideration that LXA_4_ is easily oxidized [17] and PLGA MS have the ability to preserve the biological activity of lipid mediators [18–23]. We demonstrated that LXA_4_-MS applied to rat skin ulcers were more effective than soluble LXA_4_ in accelerating the wound healing process. We propose LXA_4_-MS as an innovative and viable option to treat skin ulcers.

## Materials and methods

### Microparticle preparation

LXA_4_-MS and Un-MS were prepared using the single emulsion oil/water (o/w) method [18]. Briefly, 250 μl of LXA_4_ solution (initial concentration: 100 μg/ml) (Cayman Chemical Company, USA) was added to a solution containing 10 ml of methylene dichloride and 100 mg of PLGA 50:50 (lactic/glycolic acid) (Boehringer Ingelheim, Germany). This organic phase was afterwards mixed into an aqueous phase containing surfactant (poly (vinyl alcohol) PVA 3% w/v (Aldrich Chemicals, USA). After they were stirred for 4 h at 22°C in an RW20 IKA homogenizer (IKA Labortechnik, Germany), the microparticles were washed two times with deionized water, suspended in 1 ml of PBS, and stored at -20°C. After, LXA_4_-MS were lyophilized for 24 h and then stored at -80°C. Un-MS were prepared with the same protocol without the addition of LXA_4_.

### Microparticle characterization

Particle diameter was characterized using the LS 13 320 Laser Diffraction Particle Size Analyzer (Beckman Counter, USA), and zeta potential was determined using Nano Zeta Sizer (Malvern Instruments, England), employing 5 mg of the MS samples suspended in 1 ml of PBS. MS covered with gold were used to analyze morphology by scanning electron microscopy (SEM) using the Evo 50 (Zeiss, England). To determine encapsulation efficiency, 10 mg of LXA_4_-MS was dissolved in 1 ml of acetonitrile, vortexed to complete dissolution, evaporated for 2 h using Speed Vacuum (Eppendorf, Germany), and suspended in EIA buffer for LXA_4_ quantification using enzyme immunoassay EIA Kit EA45 (Oxford Biomedical Research, USA). Encapsulation efficiency was calculated as described [18]. To determine whether MS were contaminated by LPS, a limulus amebocyte lysate test (LAL) (Pierce Biotechnology, USA) was performed, which showed an insignificant amount of LPS in the MS. The release kinetics of LXA_4_ was monitored *in vitro*. LXA_4_ release from MS was assessed using a modified Franz-type diffusion cell (Microette; Hanson Research, USA) [18] and cellulose acetate membrane with a 0.45-μm pore size (Fisher, USA) that was placed between the sample and the receptor chamber. LXA_4_-MS were suspended in 300 μl of saline buffer (PBS; pH 7.4) and placed on top of the membrane that is the donor compartment. Samples (1 ml) were collected at 0.5, 1, 3, 6, 9, 12, 24, and 48 h from a receiving compartment containing PBS/ethanol (50:50, v/v). The samples were analyzed using a reverse phase C18 Ascentis Express column 10 cm x 2.1 mm, 2.7 μm (Sigma-Aldrich, USA) and binary gradient of 0.002% acetic acid in water/acetonitrile 7:3 solution (A) and acetonitrile/2-propanol solution (B) at a constant flow rate of 0.6 ml/min. Concentration of lipids mediators were calculated (Triple TOF 5600+ LC-MS/MS System; Sciex, USA) from linear calibration curves by MRM^HR^ experiments. The experimental data were evaluated with MultQuant 3.0 (Sciex, USA).

### Fibrin sealant (glue) preparation

Production of fibrin sealant was adapted from the Skin Cell Culture Laboratory, Universidade Estadual de Campinas (UNICAMP), Campinas, São Paulo, Brazil, as previously proposed [24–26].

### Animals and wound injury

Wistar rats (*Rattus norvegicus*) (105 adult male, weight: 180–220 g; age: 6–7 weeks old) from the Bioterium of Campus of Ribeirão Preto, Ribeirão Preto, São Paulo, Brazil were used. Animals were singly housed and maintained on a light/dark cycle with free access to food and water. Rats were divided into four groups (n = 15): untreated (Vehicle—PBS/glue); treated with Un-MS; soluble LXA_4_; LXA_4_-MS. Rats were anesthetized intraperitoneally (i.p.) with ketamine (80 mg/kg) and xylazine (15 mg/kg), and thereafter shaved and cleaned with 70% ethanol. Two excisions were made on the dorsal cervical region of the rats with a sterile histological (biopsy) punch of 1.5 cm diameter (Stiefel Laboratories, Germany). For the treatment, wounds were treated with either 10 mg of MS (Un-MS or LXA_4_-MS) or soluble LXA_4_, and the wounds were sealed with fibrin glue every 3 days, beginning on the day of wound induction (day 0). For the untreated group, PBS and fibrin glue were used. Rats were euthanized in a CO_2_ chamber and wounds plus surrounding areas were cut with a sterile histological punch. On the day of collection, one ulcer was stored at -80°C for later use in cytokine analyses, MPO measurements, and qRT-PCR. The other ulcer was used for histological analysis (H/E and Picro Sirius Red). In the experiment using WRW4 (specific antagonist of ALX receptor) (WRW4, Formyl Peptide Receptor-Like 1 (FPRL1) antagonist (Aaspec EGT Group, USA), the following groups were included: skin ulcers treated with WRW4 (25 μg of the compound in 25 μl of PBS per wound), skin ulcers treated with WRW4 + LXA_4_-MS (WRW4 applied 10 minutes before MS application, 10 mg of LXA_4_-MS), and skin ulcers treated with LXA_4_-MS (10 mg). The animals did not receive anti-inflammatory drugs commonly used to promote analgesia because these compounds may interfere with wound healing and LXA_4_ actions. All animal experiments were conducted in accordance with the Ethical Principles in Animal Research adopted by the National Council for the Control of Animal Experimentation (CONCEUA), Brazil (Process n° 016/2014-1). This study was approved by Animal Care and Use Committee of the School of Pharmaceutical Sciences of Ribeirão Preto (CEUA-FCFRP) (Process: n° 016/2014-1).

### Wound-healing index

Wound-healing was analyzed using microscopic images of skin ulcers at days 0, 2, 7, and 14 for all groups. Diameters of wound areas were calculated using ImageJ (NIH, USA). The wound healing index was calculated as per a previous study [27]. The wound-healing index ranged from 0 to 1, with 0 being a circular 1.5 cm wound and 1 being a completely closed wound.

### Histological analysis

Collected wound tissues were fixed in 4% phosphate-buffered formaldehyde for 24 h and then prepared according to standard protocols for staining with hematoxylin and eosin (H/E) or Picro Sirius Red. The sections were examined in a blinded fashion using a digital camera LEICA DFC 280 (Leica, Germany) attached to a light microscope LEICA DM 4000B (Leica, Germany). Cell counting and angiogenesis were performed using ImageJ with the Cell Counter plug-in (NIH, USA). Collagen deposition was analyzed using the ImageJ software with the Color Deconvolution plug-in.

### Cytokines, MPO, and NAG measurements

A total of 100 mg of tissue was homogenized in 1 ml of PBS, centrifuged (3,000 × *g*/10 min, 4°C), and supernatants were used to quantify IL-6, TGF-β, TNF-α, IL-1β, and VEGF by Enzyme-Linked Immunosorbent Assay (ELISA; R&D Systems, USA), MPO for neutrophil quantification as described before [27,28], and *N*-acetylglucosaminidase (NAG) for macrophage measurement [29].

### RNA Extraction and qRT-PCR

RNA was extracted using 30 mg of tissue and Ambion PureLink RNA Mini Kit according to the manufacturer’s instructions (Life Technologies, USA). Relative quantification was performed using the ΔΔCt method. For qRT-PCR, the *ALX* primers used were as follows: forward primer, 5′-TGTTGGGCCCTGGATTTTAGC-3′; and reverse primer, 5′-TGTTACCCCAGGATGCGAAGTT-3′ (antisense: nt 532–553) for LXA_4_ receptor (*Rattus norvegicus*). There were no repetitive sequences detected. For *MMP8* qRT-PCR analysis, we used commercially available TaqMan primers and probes for the TaqMan Gene Expression Assay (Applied Biosystems, USA).

### Statistical analysis

Statistical differences between groups were determined using one-way ANOVA followed by Newman-Keuls post-hoc test or Student’s *t*-test. Statistical significance was determined as p < 0.05. Data in all figures represent means ± SEM, except for the microparticles diameter and residual charges that are presented as mean ± SD.

## Results

### Characterization of LXA_4_-MS and Un-MS

**Fig 1:**
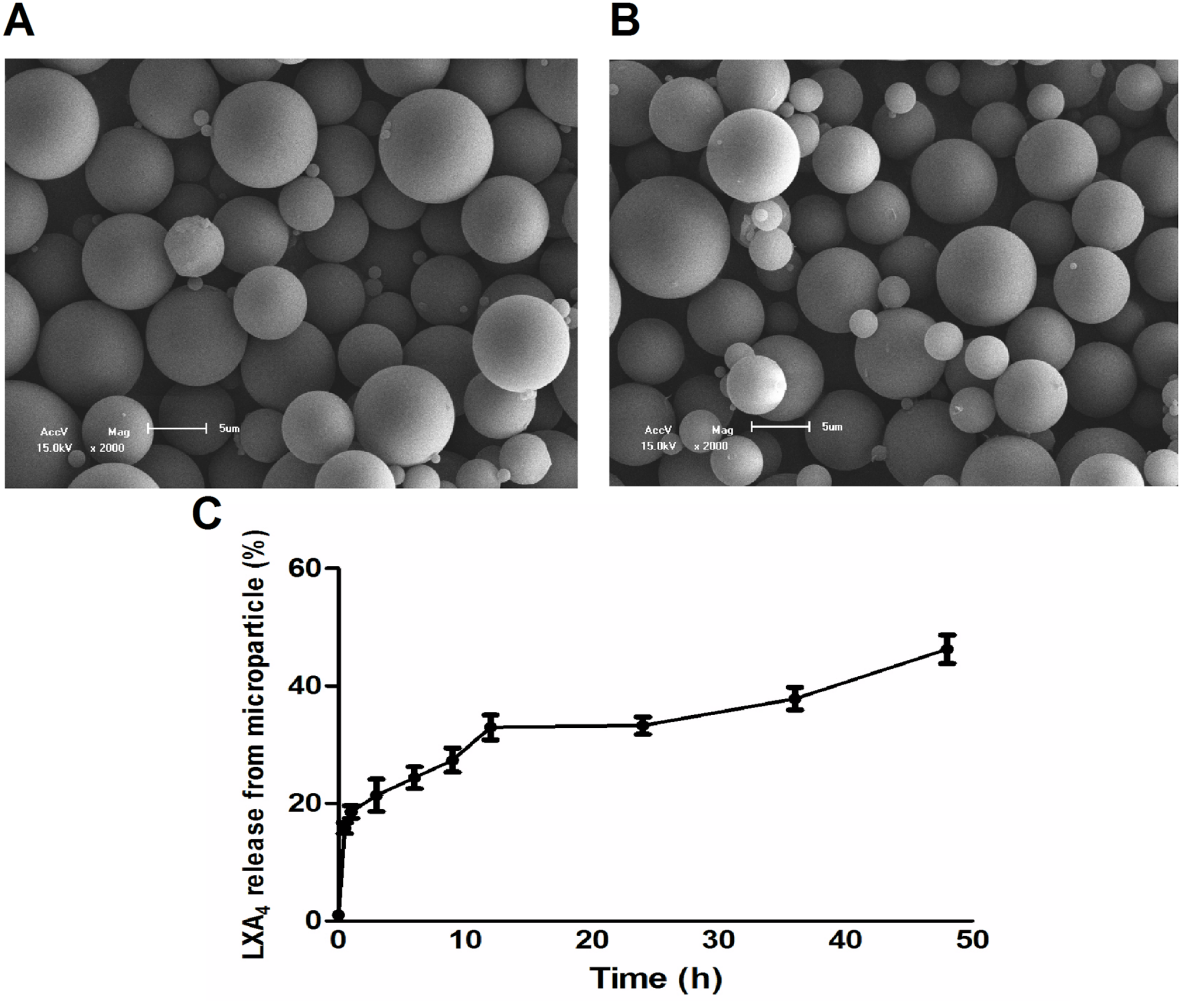
Scanning electron microscopy (SEM) of microparticles and *in vitro* release of LXA_4_ from MS. Representative images (2,000×) of (A) Unloaded and (B) LXA_4_-MS morphologies. (C) *In vitro* cumulative release of LXA_4_ from LXA_4_-MS. LXA_4_ concentration was determined by mass spectrometry over 48 h. Data are representative of two batches.

Lyophilized LXA_4_-MS and Un-MS were dispersed in deionized water, and their size and residual charges were measured. The average diameter was 5.4 μm (± 5.6 μm SD) and 3.9 μm (± 4.4 μm SD) for LXA_4_-MS and Un-MS, respectively. Analysis of residual charges demonstrated that the molecule, which was encapsulated, did not significantly alter the charges of the polymers. Electrokinetic potential in colloidal dispersions (zeta) was -1.13 mV (± 9.6 mV SD) and -4.56 mV (± 4.8 mV SD) for LXA_4_-MS and Un-MS, respectively. The high levels of variation in both residual charges and diameter are expected, since during the preparation process, millions of particles are generated with an acceptable range of diameter and electric charges. However, the majority of MS have the appropriated size to penetrate the skin corneum, and negative charge to interact with cell membranes. SEM of Un-MS and LXA_4_-MS showed the presence of spherical and uniform surfaces (Fig 1A and 1B). Encapsulation efficiency (the amount of LXA_4_ encapsulated per 10 mg of lyophilized MS) was evaluated using an immunoassay (EIA) kit. We found 800-ng/10 mg or an encapsulation efficiency of 32%. This finding was lower than we observed for leukotriene B_4_ (LTB_4_) or prostaglandin E_2_ (PGE_2_), which had encapsulation efficiencies of 50% and 75%, respectively [18,19,22]. The *in vitro* release rates from PLGA MS were evaluated for up to 48 h, and the release profile of LXA_4_ is shown in Fig 1C. The result demonstrated that LXA_4_ released from the MS was sustained. Despite the lower encapsulation efficiency, these data showed that PLGA is a suitable strategy for use as a delivery system for lipid mediators.

### LXA_4_-MS reduced neutrophil chemotaxis and accelerated wound closure

**Fig 2:**
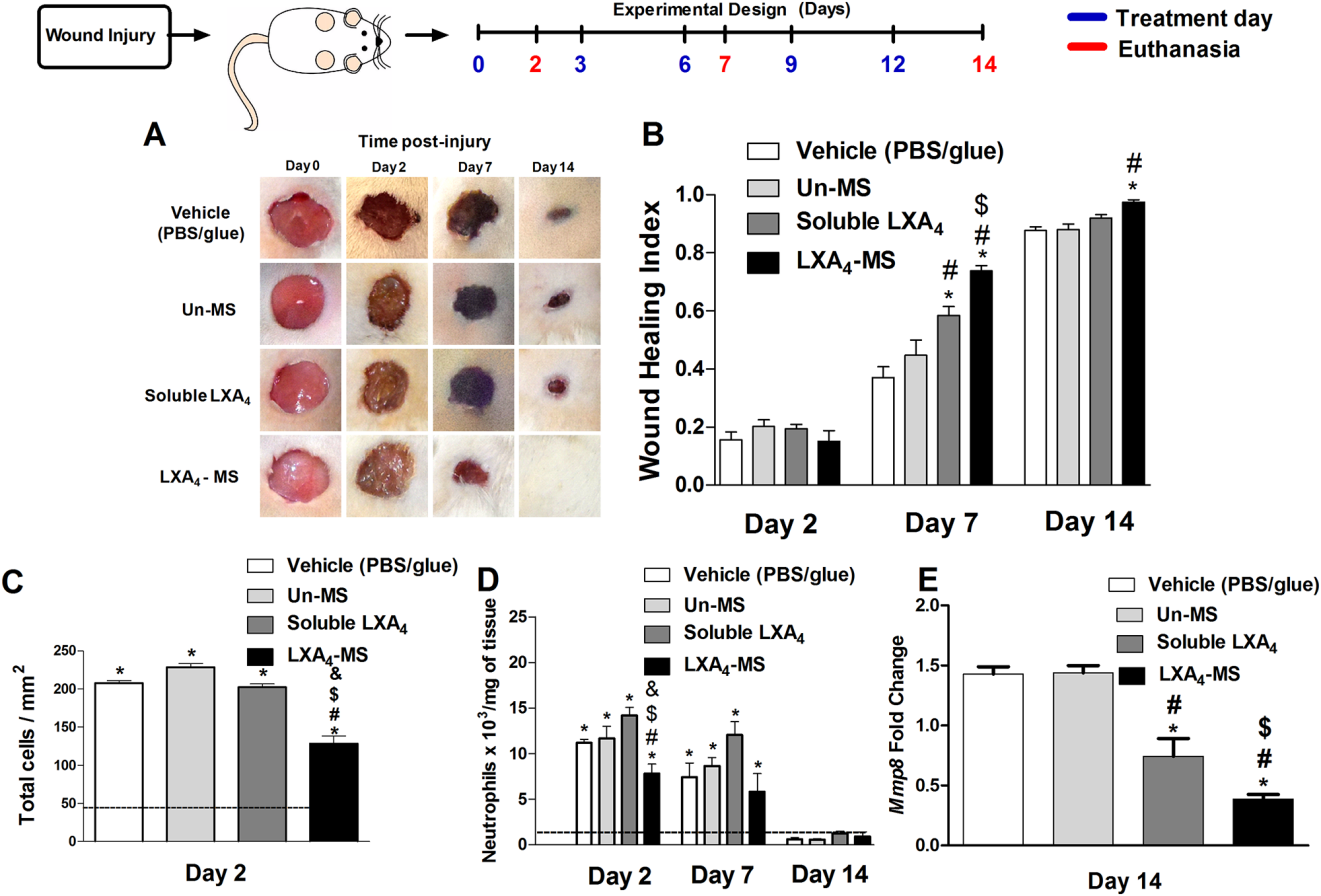
Topical application of LXA_4_-MS to skin ulcers accelerated wound closure and attenuated neutrophil chemotaxis. (A) Representative images of 1.5 cm dorsal wounds were collected on days 0, 2, 7, and 14 for the following groups: control (vehicle—PBS/glue), Un-MS, soluble LXA_4_, and LXA_4_-MS. (B) Wound healing index values for the groups outlined in (A). Index values range from 0 to 1, where a value of 0 indicates the original wound, and a value of 1 represents a completely closed wound. Values are means ± SEM (n = 10 ulcers/group). One-way ANOVA was done to determine statistical significance (*p* < 0.05), which is as follows: *, soluble LXA_4_ or LXA_4_-MS *vs*. vehicle (PBS/glue); ^#^, LXA_4_-MS or soluble LXA_4_ *vs*. Un-MS; and ^$^, LXA_4_-MS *vs*. soluble LXA_4_. (C) ImageJ software was used to count inflammatory cells on day 2 in at least 12 random optical 400× fields per group. (D) Neutrophil accumulation (represented as bars) as measured by MPO. Values are means ± SEM (n = 5 wounds/group). One-way ANOVA was done to determine statistical significance (*p* < 0.05), which is indicated as follows: *, demonstrated significant increase compared to normal tissue (dashed line); ^#^, soluble LXA_4_ or LXA_4_-MS *vs*. Vehicle (PBS/glue); ^$^, LXA_4_-MS or soluble LXA_4_ *vs*. Un-MS; and &, LXA_4_-MS *vs*. soluble LXA_4_. (E) qRT-PCR was performed to assess *MMP8* mRNA transcript abundance in skin ulcers collected on days 2, 7, and 14 from the vehicle (PBS/glue), Un-MS, soluble LXA_4_, and LXA_4_-MS groups. Data represent means ± SEM (n = 5 ulcers/group). One-way ANOVA was done to determine statistical significance (*p* < 0.05) and indicated as follows: *, soluble LXA_4_ or LXA_4_-MS *vs*. Vehicle (PBS/glue); ^#^, LXA_4_-MS or soluble LXA_4_ *vs*. Un-MS; and ^$^, LXA_4_-MS *vs*. soluble LXA_4_.

Treatment of skin ulcers with LXA_4_-MS accelerated wound closure beginning at 7 days post injury (Fig 2A and 2B) as compared to wounds treated with PBS/glue (vehicle), Un-MS, and soluble LXA_4_. Particularly on day 7, ulcers receiving only fibrin glue and PBS presented only 39% closure of the initial ulcer diameter. Soluble LXA_4_ and Un-MS improved wound healing by inducing 60% and 45% closure of wounds, respectively. Treatment with LXA_4_-MS induced closure of 80% of initial ulcers. Interestingly, on day 14, only the induced ulcers treated with LXA_4_-MS were completely healed. Next, we evaluated leukocyte recruitment to the wound site using two distinct strategies: histological analysis and myeloperoxidase (MPO) measurement. We observed that the number of total cells on wounds treated with LXA_4_-MS was reduced in comparison to the other groups (Fig 2C and S1A Fig). Assessing tissue MPO and matrix metalloproteinase-8 (*MMP8)* mRNA abundance (Fig 2D and 2E), we confirmed that neutrophils were lower in LXA_4_-MS wounds at days 2 and 7 compared to that in the control, Un-MS, and soluble LXA_4_ groups (although soluble LXA_4_ also decreased neutrophil recruitment). These data demonstrated that LXA_4_-MS possessed higher inflammatory resolution activity and was therefore able to expedite wound healing. Moreover, the strategy of encapsulating LXA_4_ in PLGA efficiently preserved its biological function.

### LXA_4_-MS affected pro and anti-inflammatory cytokine production

**Fig 3:**
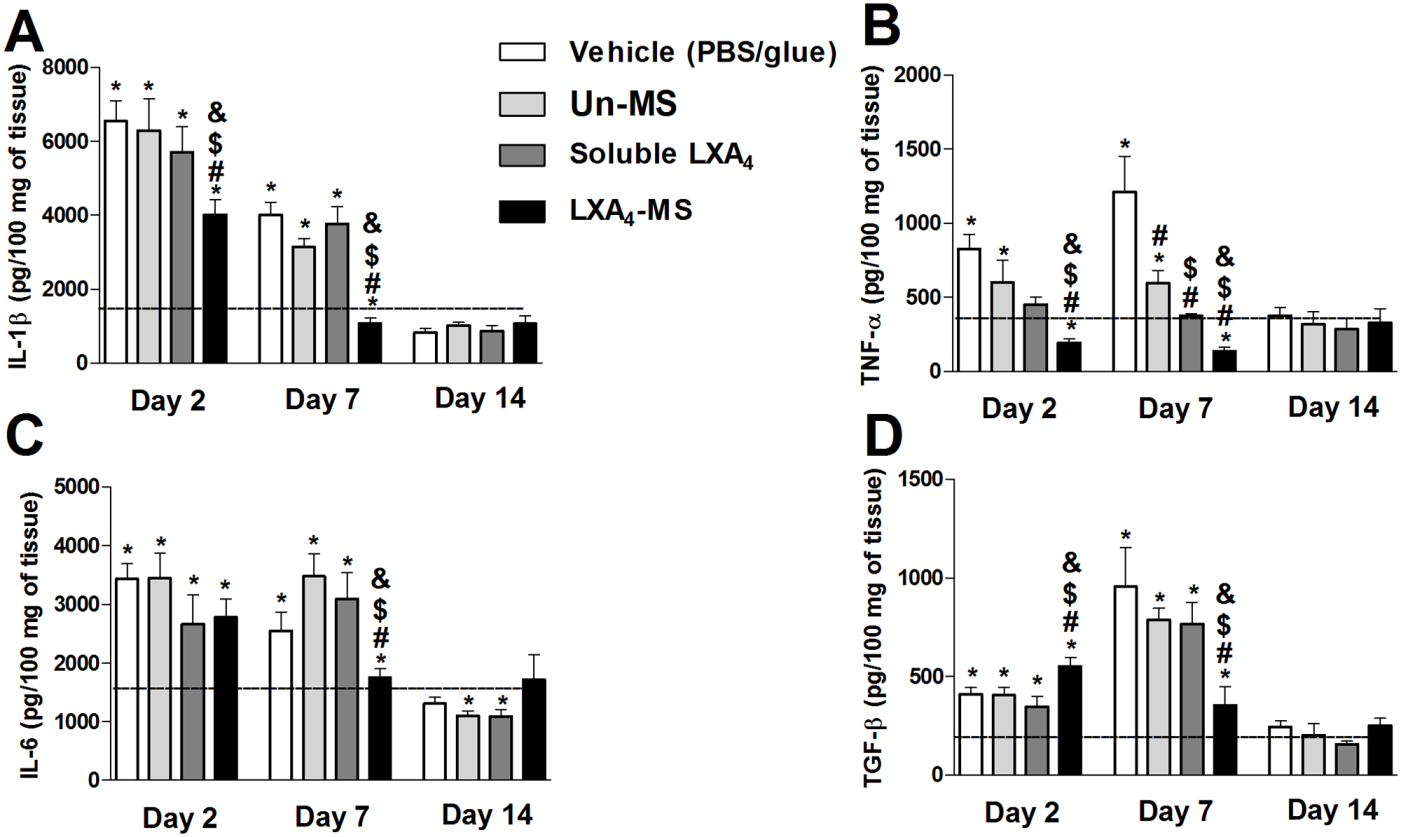
LXA_4_-MS modulated cytokines generation in the skin. Skin ulcer tissues collected on days 0, 2, 7, and 14 from the control (vehicle—PBS/glue), Un-MS, soluble LXA_4_, and LXA_4_-MS groups were homogenized to assess (A) IL-1β, (B) TNF-α, (C) IL-6, and (D) TGF-β production by ELISAs. Data (represented as bars) represent means ± SEM (n = 5 ulcers/group). One-way ANOVA was done to determine statistical significance (*p* < 0.05) and indicated as follows: *, demonstrated significant differences compared to normal tissues (dashed line); ^#^, soluble LXA_4_ or LXA_4_-MS *vs*. Vehicle (PBS/glue); ^$^, LXA_4_-MS or soluble LXA_4_ *vs*. Un-MS; and ^&^, LXA_4_-MS *vs*. soluble LXA_4_.

We investigated the effects of all treatments on cytokine production in skin ulcers (Fig 3). We observed that LXA_4_-MS treatment reduced the production of IL-1β, TNF-α, and IL-6 at days 2 and 7, although with variable intensity. No differences were observed after 14 days post skin injury (Fig 3A, 3B and 3C). Interestingly, LXA_4_-MS treatment first induced an increase in TGF-β production at day 2 and a decrease in TGF-β production at day 7 (Fig 3D). In ulcers from the other groups, we observed little or no effect on the production of cytokines. At day 14, cytokine concentrations resembled levels found in non-injured skin (day 0) and were thus used to obtain a basal level of cytokines, serving as internal controls.

### LXA_4_-MS promoted and increased deposition of collagen, angiogenesis, and vascular endothelial growth factor (VEGF) production

**Fig 4:**
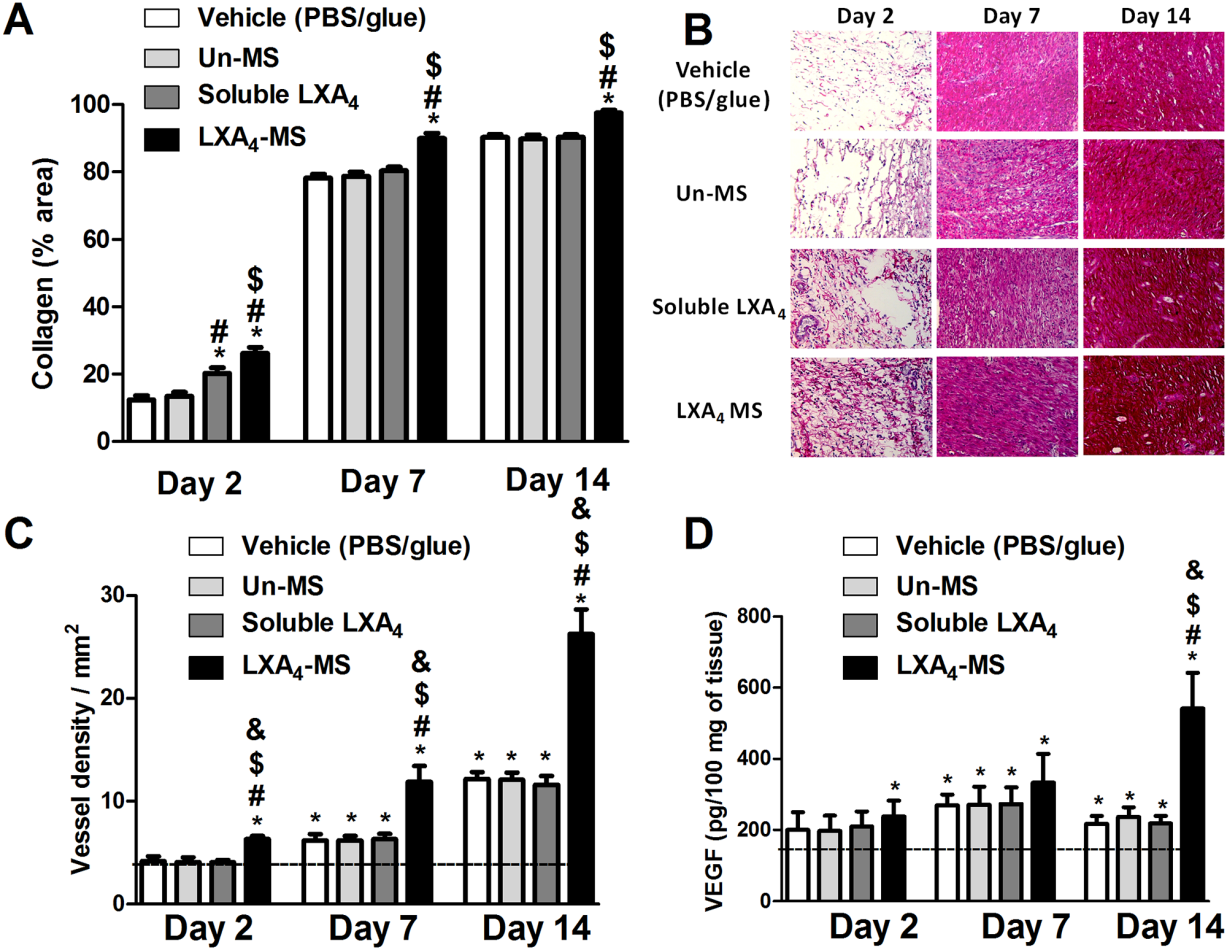
LXA_4_-MS increased collagen deposition and angiogenesis and affected VEGF production. (A) Collagen deposition was measured using the ImageJ software with the Color Deconvolution plug-in, which measured densitometry in at least 12 random 400× fields in all groups, at days 2, 7 and 14. Percentages of collagen deposition are represented as means ± SEM (n = 6 ulcers/group). One-way ANOVA was used to determine statistical significance (*p* < 0.05) and is indicated as follows: *, soluble LXA_4_ or LXA_4_-MS *vs*. Vehicle (PBS/glue); ^#^, LXA_4_-MS or soluble LXA_4_ *vs*. Un-MS; and ^$^, LXA_4_-MS *vs*. soluble LXA_4_. (B) Photomicrographs of wounds stained with Picro Sirius Red (200×) show collagen deposition at days 2, 7, and 14. (C) Histogram showing quantitative analysis of vascular density using the ImageJ software with the Cell Counter plug-in on 200× images. One-way ANOVA was performed to determine statistical significance (*p* < 0.05), which is indicated as follows: *, significant VEGF increase as compared to normal tissue (dashed line); ^#^, soluble LXA_4_ or LXA_4_-MS *vs*. Vehicle (PBS/glue); ^$^, LXA_4_-MS or soluble LXA_4_ *vs*. Un-MS; and ^&^, LXA_4_-MS *vs*. soluble LXA_4_. (D) VEGF was quantified in all groups at days 2, 7 and 14 (represented as bars) in wounds via ELISAs as proxy to blood vessel density. Values are means ± SEM (n = 6 wounds/group). One-way ANOVA was performed to determine statistical significance (*p*< 0.05), which is indicated as follows: *, significant VEGF increase as compared to normal tissue (dashed line); ^#^, soluble LXA_4_ or LXA_4_-MS *vs*. Vehicle (PBS/glue); ^$^, LXA_4_-MS or soluble LXA_4_ *vs*. Un-MS; and ^&^, LXA_4_-MS *vs*. soluble LXA_4_.

Since we observed that LXA_4_-MS increased wound healing, we investigated its impact on the elements important for skin restoration, such as collagen deposition and angiogenesis. We observed that LXA_4_-MS enhanced collagen deposition beginning at day 2 and peaking at day 14 (Fig 4A). The enhancement of collagen deposition was confirmed by histological analysis. LXA_4_-MS induced massive deposition of collagen on skin ulcers; although with less intensity, soluble LXA_4_ also induced collagen deposition (Fig 4B). The number of blood vessels in histological sections stained by hematoxylin/eosin (H/E) and VEGF quantification in skin ulcers were additionally evaluated. As expected, LXA_4_-MS stimulated massive neovascularization beginning on day 2 (Fig 4C and S1B Fig) and peaking at 14 days. However, an increase in VEGF was observed only after 14 days, the last day of observation (Fig 4D). Soluble LXA_4_ and Un-MS did not induce blood vessel formation nor increase in VEGF levels.

### LXA_4_-MS induced increase of macrophages and IL-4 in the skin

**Fig 5:**
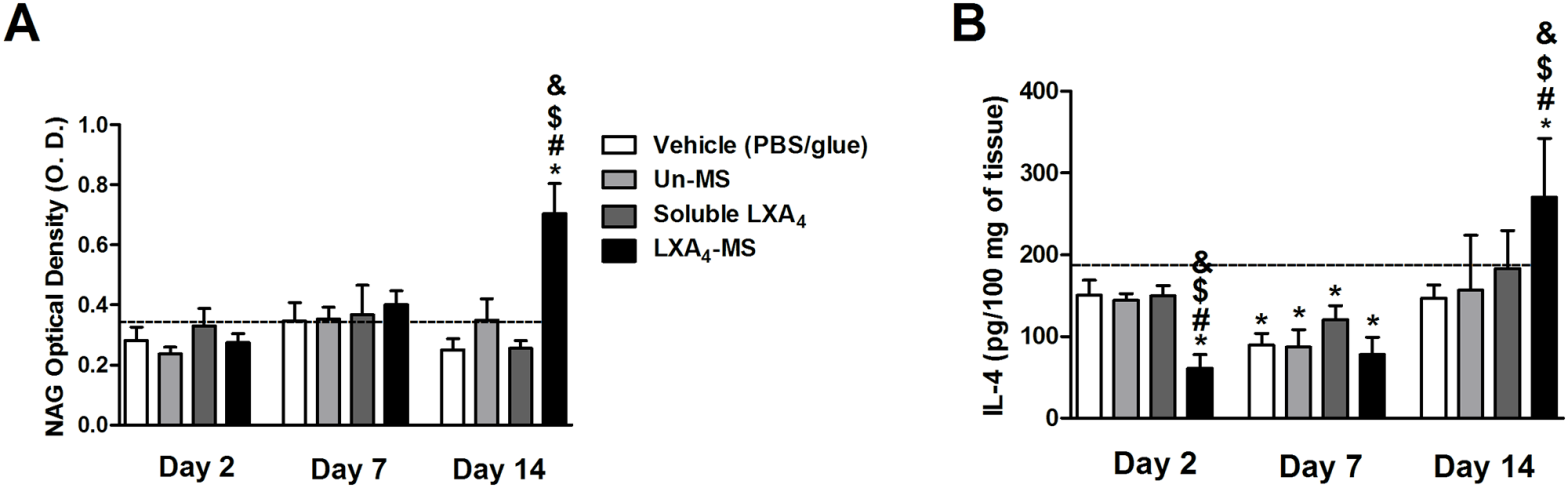
LXA_4_-MS increased macrophages and IL-4 production. (A) NAG (OD values) was quantified in skin ulcers collected on days 2, 7, and 14 from the vehicle (PBS/glue), Un-MS, soluble LXA_4_, and LXA_4_-MS groups (n = 5 ulcers/ group). (B) ELISAs were used to measure IL-4 production in skin ulcers collected on day 14 from the vehicle (PBS/glue), Un-MS, soluble LXA_4_, and LXA_4_-MS groups. Basal levels were also assessed. Data represent means ± SEM (n = 5 ulcers/ group). One-way ANOVA was done to determine statistical significance (*p* < 0.05) and indicated as follows: *, demonstrated significant differences compared to normal tissues (dashed line); ^#^, LXA_4_-MS *vs*. Vehicle (PBS/glue); ^$^, LXA_4_-MS *vs*. Un-MS; and ^&^, LXA_4_-MS *vs*. soluble LXA_4_.

Type II macrophages are known to release IL-4 [30], which regulates scar formation. Thus, we estimated macrophage infiltration by NAG quantification, a marker of these leukocytes [31]. We observed that LXA_4_-MS treated lesions exhibited a significant increase on macrophage infiltration at day 14 (Fig 5A). We also observed enhanced IL-4 production, which was probably released by infiltrating macrophages (Fig 5B), as described before [30]. Un-MS and soluble LXA_4_ did not increase NAG and IL-4 production.

### LXA_4_-MS increased ALX receptor expression and treatment with a specific ALX receptor antagonist reversed LXA_4_-MS wound healing capabilities

**Fig 6:**
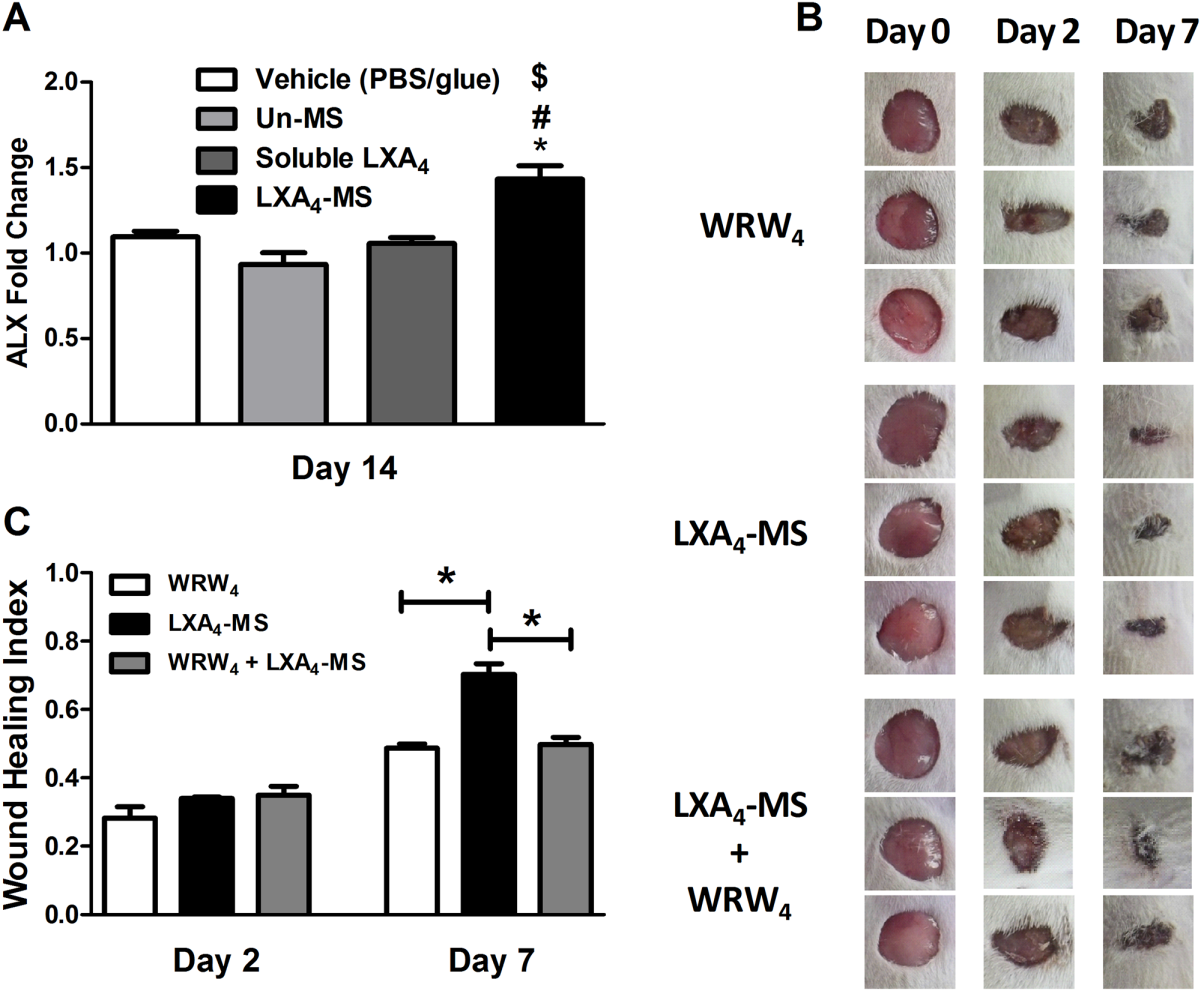
WRW4, a selective LXA_4_ receptor antagonist, reversed wound healing properties of LXA_4_-MS. (A) qRT-PCR analysis was performed to assess the LXA_4_ receptor *ALX* mRNA’s abundance in skin ulcers collected on days 2, 7, and 14 in the control (vehicle—PBS/glue), Un-MS, soluble LXA_4_, and LXA_4_-MS groups. Data represent means ± SEM (n = 5 ulcers/group). One-way ANOVA was done to determine statistical significance (*p* < 0.05), which is indicated as follows: *, LXA_4_-MS *vs*. Vehicle (PBS/glue); ^#^, LXA_4_-MS *vs*. Un-MS; and ^$^, LXA_4_-MS *vs*. soluble LXA_4_. (B) Representative images of 1.5 cm dorsal wounds at days 2 and 7, with day 0 images serving as pre-injury images, are presented for the following groups: WRW4 (25 μl per animal—from a main peptide solution of 1 mg/ml), WRW4 + LXA_4_-MS (WRW4 applied 10 minutes before MS application– 10 mg of LXA_4_-MS), and LXA_4_-MS (10 mg). (C) Wound healing index values for the groups outlined in (B). Index values range from 0 to 1, where a value of 0 indicates the original wound, and a value of 1 represents a completely closed wound. Data represent means ± SEM (n = 9 ulcers/group); One-way ANOVA was done to determine statistical significance (*p < 0.05).

To investigate whether LXA_4_ released from the microparticles induced wound healing via its own receptor ALX, we assessed ALX expression via qRT-PCR on day 14; the healed skin appeared to be similar to the intact skin. Our results demonstrated that at 14 days post lesion, remarkable up regulation occurred in the mRNA ALX receptor (Fig 6A). As seen in Fig 2A, 7 days after injury, LXA_4_-MS promoted 80% wound closure compared to the initial wound. Therefore, day 7 was chosen for the analysis of the role of LXA_4_ receptor. We treated skin lesions with LXA_4_-MS in the presence or absence of WRW4, a specific LXA_4_ receptor antagonist. WRW4 treatment reversed LXA_4_-MS ability to efficiently heal skin ulcers as assessed microscopically and via the wound healing index values (Fig 6B and 6C). Taken together, these results demonstrated that LXA_4_ released from the microparticles improved wound healing expressly through its ALX receptor.

## Discussion

After injury, wound healing elicits a coordinated cascade of intracellular and intercellular events to restore homeostasis and tissue integrity [1]. It is a dynamic process divided into distinct phases [1]. Crucial to healing and cell recruitment are cytokines and lipid mediators production, collagen deposition, and blood vessel formation [1,2]. Among the soluble mediators involved in the healing process, LXA_4_ is a key factor, especially for cornea healing [15]. This mediator is derived from AA metabolism [8,32] and a potent inhibitor of neutrophil recruitment to the site of inflammation and regulator of leukocyte chemotaxis, and activation [9–13]. Macrophages are then activated to transmigrate and to promote phagocytosis of apoptotic PMN, creating an environment conducive for wound-healing [15]. However, the role of LXA_4_ in skin regeneration after injury remains unknown.

LXA_4_ is an unstable mediator [17], a feature that impairs its use for treating inflammatory diseases or tissue lesions. We took advantage of our expertise in lipid mediator encapsulation in PLGA microparticles in order to preserve its wound healing activities and provide controlled release [18,19,22]. Using the single emulsion oil/water (o/w) method [18], we prepared and characterized the Un-MS and LXA_4_-MS to ensure appropriate size 5.4 μm (± 5.6 μm SD) and 3.9 μm (± 4.4 μm SD) for LXA_4_-MS and Un-MS] respectively, and charge (-1.13 mV and -4.56 mV for LXA_4_-MS and Un-MS respectively) needed to interact with cells (Fig 1). According to Jalon’s study, microparticles with a size of around 5 μm are appropriate to penetrate into the stratum corneum, reaching the epidermis and promoting sustained drug release into the skin [33]. The amount of PLGA microparticles loaded with LXA_4_ (10 mg of MS containing 800 ng of LXA_4_) used to treat the wound in our study was selected based on literature data, demonstrating that 1 μg of soluble LXA_4_ accelerated epithelial wound healing in the cornea [15]. To pursue this aim, we used a well-characterized model to study skin healing in rats [34,35]. After inducing skin lesions, ulcers were treated with each PBS, Un-MS, soluble LXA_4_, or LXA_4_-MS and then covered with fibrin glue. The healing process was assessed 2, 7, and 14 days later. We showed that using PLGA microparticles to encapsulate LXA_4_ preserved its biological activities, as evidenced by our calculated wounding healing index values (Fig 2A and 2B). Remarkably, lesions treated with LXA_4_-MS were completely closed after 14 days. Soluble LXA_4_ has been shown to reduce neutrophil infiltration in the cornea [15], and our data confirmed that LXA_4_-MS also reduced neutrophil infiltration in the wound site at 2 and 7 days as per the decrease in MPO (Fig 2D) and *MMP8* mRNA (Fig 2E), which are both markers of neutrophil infiltration [36,37]. Decrease in neutrophil infiltration has been described as beneficial for wound healing. A recent study demonstrated that the ‘neutrophil extracellular trap’ (NET) facilitated matrix collagen degradation, impairing wound-healing [38]. Our data suggested that LXA_4_-MS attenuated neutrophil chemotaxis (Fig 4A and 4B); this effect allows collagen deposition, as previously demonstrated by others [38,39]. As expected, among the other groups, only soluble LXA_4_ increased the wound-healing index at 7 days and reduced *MMP8* mRNA at 14 days (Fig 2B and 2E). However, the soluble mediator minimally affected lesion closure, probably due to its intrinsic instability and thus loss in its biological properties. It’s important to note that MMP8 was not measured days 2 and 7 owing to low tissue recovery and intense inflammatory cell infiltration, which may lead to false-positive results.

LXA_4_ mediates the delicate balance among inflammatory, anti-inflammatory and regulatory cytokines, which modulate tissue regeneration [15]. Particularly, LXA_4_ inhibits NF-κB and AP-1 and consequently decreases pro-inflammatory cytokines production [7]. Simultaneously, LXA_4_ activates the transcriptional factor PPAR-γ, generating a resolutive profile [40]. Our results demonstrated that the acceleration of wound closure and the reduction in neutrophil recruitment induced by LXA_4_-MS treatment correlated with altered cytokine profiles. We also observed that LXA_4_-MS application reduced the concentration of the inflammatory cytokines IL-1β, TNF-α, and IL-6. Previous works have shown that such reduction is accompanied by increased TGF-β, an anti-inflammatory cytokine, and collagen deposition [41]. LXA_4_-MS increased collagen deposition and blood vessel density in coordination with VEGF release (Fig 4C and 4D; S1B Fig). Moreover, at the site of the lesion, LXA_4_-MS increased macrophages and IL-4. These findings suggested that the accumulation of type II macrophages, sources of IL-4 a regulatory cytokine, is mediates LXA_4_ resolution properties [30]. Furthermore, wound-healing may function to mediate recruitment of macrophages to infection sites in order to promote phagocytosis of apoptotic PMN and clearance and remodeling of the tissue [14].

Finally, using a selective antagonist of LXA_4_ receptor, WRW4 [42], we demonstrated that the beneficial effects of LXA_4_-MS treatment were indeed mediated by the interaction of LXA_4_ specifically to its receptor ALX (Fig 6) and not due to unspecific effects of the PLGA microparticles. The participation of ALX receptor in an *in vitro* model of regeneration was described recently in a study using soluble LXA_4_ [43]. We observed that 14 days after injury (the skin regeneration period), an increase in ALX mRNA occurred. This finding suggested that, at least within this timeframe, endogenous LXA_4_ is not produced in the necessary quantity to close the lesion. However, we could not rule out the possibility of LXA_4_ being released beyond 14 days, the same period of spontaneous lesion closure. Also, the relevance of LXA_4_ to eye tissue regeneration has been demonstrated [15]. Our results suggested that LXA_4_ released from LXA_4_-MS accelerated skin wound-healing via its ALX receptors, findings that corroborated a previous study [7].

## Conclusions

In summary, we show the retention of LXA_4_ as a resolution, wound healer, and reparative mediator via the use of PLGA microparticles. Our study demonstrated LXA_4_-MS as an effective strategy for the treatment of skin ulcers, and it may be a viable treatment option for healing and repair.

## Supporting information

S1 Fig LXA_4_-MS affects cellular recruitment and neovascularization. **(A)** Animals were topically treated with PBS/glue, Un-MS, soluble LXA_4_ and LXA_4_-MS. At day 2, animals were euthanized, wounds were removed and paraffin-wound sections were stained with HE to evaluate inflammatory infiltrate by image analysis. The sections were photographed at 400×. The cell counting was performed using the software ImageJ, plug-in Cell Counting in at least 12 random optic fields per group. **(B)** Animals were topically treated with vehicle (PBS/glue) or LXA_4_-MS. Paraffin wound sections were stained with HE and photographed at 400×. The blood vessels were counted using the software ImageJ, plug-in Cell Counting in at least 12 random optic fields per group.
(TIF)
