# Supplementary figures and images for: Lipoxin A4 encapsulated in PLGA microparticles accelerates wound healing of skin ulcers
Source: PLoS One. 2017 Jul 28;12(7):e0182381. doi: 10.1371/journal.pone.0182381 (PMC5533323; doi:10.1371/journal.pone.0182381)

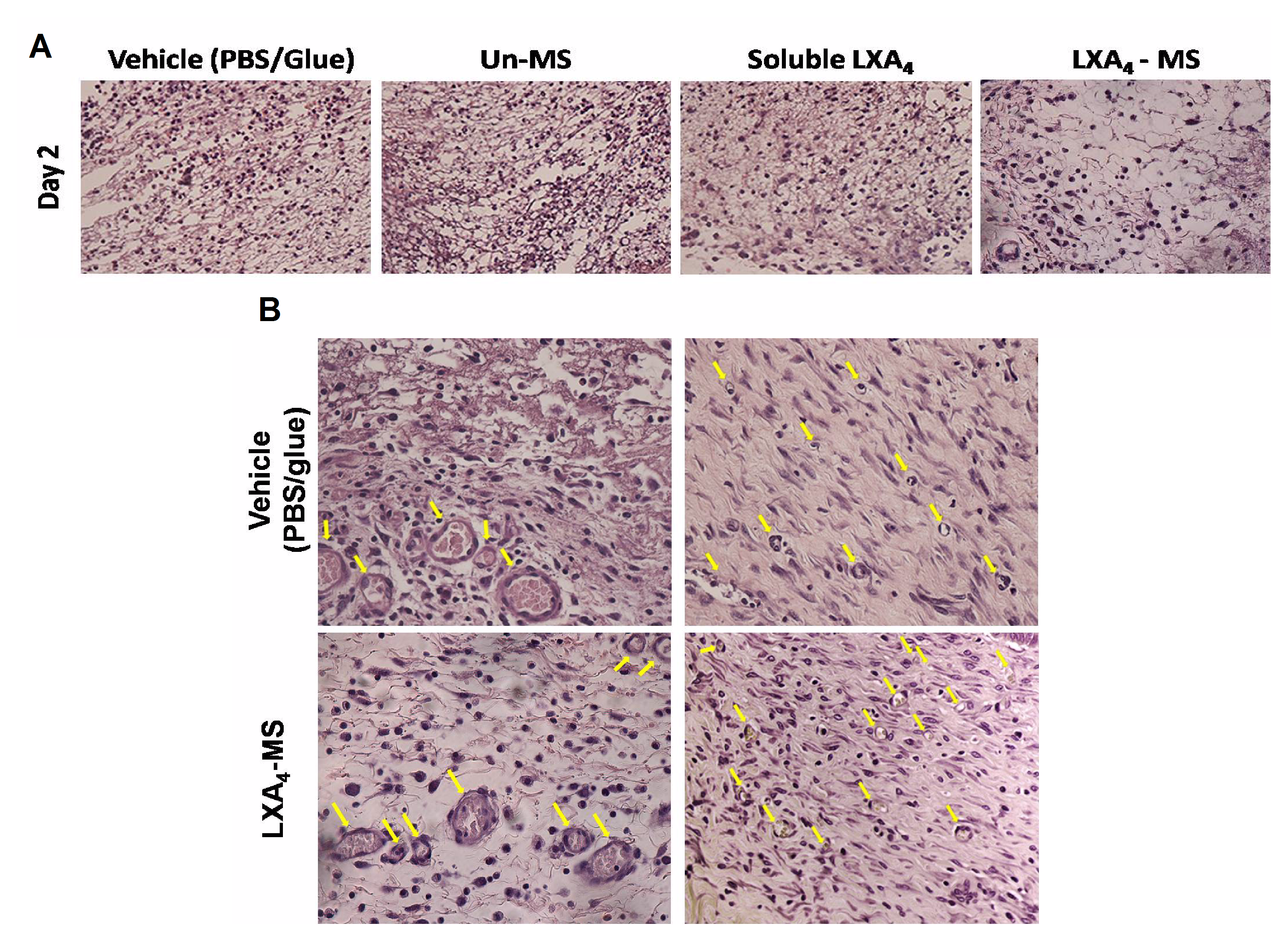

Supplement: S1 Fig — (A) Animals were topically treated with PBS/glue, Un-MS, soluble LXA4 and LXA4-MS. At day 2, animals were euthanized, wounds were removed and paraffin-wound sections were stained with HE to evaluate inflammatory infiltrate by image analysis. The sections were photographed at 400×. The cell counting was performed using the software ImageJ, plug-in Cell Counting in at least 12 random optic fields per group. (B) Animals were topically treated with vehicle (PBS/glue) or LXA4-MS. Paraffin wound sections were stained with HE and photographed at 400×. The blood vessels were counted using the software ImageJ, plug-in Cell Counting in at least 12 random optic fields per group. (TIF) [file pone.0182381.s001.tif]
